# Unraveling the intricate molecular landscape and potential biomarkers in lung adenocarcinoma through integrative epigenomic and transcriptomic profiling

**DOI:** 10.1038/s41598-025-93769-w

**Published:** 2025-03-17

**Authors:** Arnab Mukherjee, Manon Boonbangyang, Mukunthan K.S.

**Affiliations:** 1 Department of Biotechnology, Manipal Institute of Technology, Manipal Academy of Higher Education, Manipal, Manipal India; 2 Pornchai Matangkasombut Center for Microbial Genomics, Department of Microbiology, Faculty of Science, Mahidol University, Bangkok, Thailand

**Keywords:** Lung adenocarcinoma, Epigenomics, Transcriptomics, Enhancers, Regulatory networks, Mitochondrial ribosomal proteins, Biotechnology, Cancer, Computational biology and bioinformatics, Systems biology

## Abstract

Lung adenocarcinoma (LUAD) remains a leading cause of cancer-related mortalities, characterized by substantial genetic heterogeneity that challenges a comprehensive understanding of its progression. This study employs next-generation sequencing data analysis to transform our comprehension of LUAD pathogenesis. Integrating epigenetic and transcriptomic data of LUAD patients, this approach assessed the critical regulatory occurrences, identified therapeutic targets, and offered profound insights into cancer molecular foundations. We employed the DNA methylation data to identify differentially methylated CpG sites and explored the transcriptome profiles of their adjacent genes. An intersectional analysis of gene expression profiles uncovered 419 differentially expressed genes (DEGs) influenced by smoke-induced differential DNA methylation, among which hub genes, including mitochondrial ribosomal proteins (MRPs), and ribosomal proteins (RPs) such as *MRPS15*, *MRPS5*, *MRPL33*, *RPL24*, *RPL7L1*, *MRPL15*, *TUFM*, *MRPL22*, and *RSL1D1*, were identified using a network-based approach. These hub genes were overexpressed and enriched to RNA processing, ribosome biogenesis, and mitochondrial translation, which is critical in LUAD progression. Enhancer Linking Methylation/Expression Relationship (ELMER) analysis revealed transcription factor (TF) binding motifs, such as *JUN*, *NKX23*, *FOSB*, *RUNX3*, and *FOSL1*, which regulated these hub genes through methylation-dependent enhancer dynamics. Predominant hypomethylation of MRPs and RPs disrupted mitochondrial function, contributed to oxidative phosphorylation (OXPHOS) and metabolic reprogramming, favoring cancer cell survival. The survival analysis validated the clinical relevance of these hub genes, with high-expression cohorts exhibiting poor overall survival (OS) outcomes enlightened their relevance in LUAD pathogenesis and presented the potential for developing novel targeted therapeutic strategies.

**Supplementary Information:**

The online version contains supplementary material available at 10.1038/s41598-025-93769-w.

## Introduction

According to data for the year 2023, lung cancer remains a prevalent type of cancer and is expected to be a significant contributor to cancer-related fatalities globally^1,2^. Non-small cell lung cancer (NSCLC), specifically lung adenocarcinoma (LUAD), accounts for approximately 80–85% of reported cases of lung cancer^3^. Lung carcinogenesis unfolds in a sequential multistage pattern, gradually developing genetic and epigenetic alterations. Epigenetic alterations induce the development of malignant characteristics and aggressive traits in lung cancer cells and acquire resistance to conventional therapeutics by dysregulating the signaling pathways, offering new possibilities for epigenetic therapy and identifying novel therapeutic targets^4^.

Tobacco smoking has the most significant influence on the lungs, being accountable for 87% of lung cancer deaths and associated with changes in DNA methylation at CpG sites^5^. Numerous studies revealed smoking-related epigenetic alterations in lung cancer pathogenesis by modulating multiple biological pathways^6–9^. A wide range of tumors exhibit atypical methylation patterns, which can involve either increased (hypermethylation) or decreased (hypomethylation) addition of a methyl group to the cytosine. These changes are typically mediated by enzymes known as DNA methyltransferases. By implementing an epigenome-wide methylation study, we validated that tobacco smoking induces changes in DNA methylation patterns, specifically at CpG sites.

Prognostically significant aberrantly methylated enhancers form positive wiring with their target gene to impart malignant traits involving cell proliferation, angiogenesis, and invasion. The differential DNA methylation associated with smoking might be due to the activation of enhancer regions, implying that external factors have a role in the development of lung cancer through alterations in regulatory elements^10^. Enhancers govern gene expression across great distances by looping DNA and offering distant regulatory regions closer to their target gene promoters^11^. Therefore, we employed DNA methylation to determine enhancers and link enhancer status with the expression of target genes to discover transcriptional targets.

Due to advancements in high-throughput sequencing technology, the utilization of Illumina HM450k for cancer analysis has been implemented^10^. In this study, we investigated a technique for predicting enhancer-target interactions through cis-regulatory elements by combining epigenomic and transcriptomic data from a substantial collection of primary tumor samples. This approach allowed us to identify target genes regulated explicitly by enhancers with differential methylation patterns in LUAD.

## Methodology

### Data retrieval

The methylation and transcriptome profiling data of primary tumor (TP) and solid tissue normal (NT) samples of LUAD patients were accessed and downloaded from the Illumina Infinium HumanMethylation 450 K platform of The Cancer Genome Atlas (TCGA) database using the TCGAbiolinks package of Bioconductor (Release 3.14)^12^. An expression profiling data of cigarette smoke-induced enhancers in A549 cells and DNA methylation microarray data of lung cancer cells (GSE32867 and GSE69770) were retrieved from the Gene Expression Omnibus (GEO) database^10,13^.

### Differential methylation analysis

The retrieved methylation data of LUAD patients were subjected to analysis of the differentially methylated sites using the SeSAMe (V3.18) package of R (V4.1.2)^14^. The mean of the beta-methylation values was calculated for each patient in the TP (*n* = 473) and NT (*n* = 32) groups. The probes with NA value were excluded in the data preprocessing stage. The beta-methylation values are from 1 to 0, representing completely methylated and demethylated. The difference between the beta-methylation values of each probe from both groups was calculated. Further, the Wilcoxon test was employed by adjusting the Benjamini-Hochberg correction with a beta-value difference of 0.25 and a False Discovery Rate (FDR) *p*-value < 0.05^15^.

### Transcriptome profile analysis

The transcriptome profiling was performed over the TCGA-LUAD data to determine the change in the expression of the neighboring gene of the altered methylated sites. The retrieved data was subjected to preprocessing steps. The outliers were determined by performing an array-array intensity correlation and normalizing the RNA transcripts using the EDAseq (V2.36) package^16^. The normalized transcripts were employed for the Differential expression analysis (DEA) with a log2Fold Change (log2FC) > 1 and FDR *p*-value < 0.05 between the TP and NT samples. The RNA-seq and microarray dataset of the altered DNA methylation retrieved from GEO was analyzed using similar parameters to determine the Differentially Expressed Genes (DEGs).

### Integration of DNA methylation and transcriptomic data

The ELMER (V2.26) package was used to determine the distant regulatory enhancers of the target genes. This package enables the integration of the DNA methylation and transcriptomic data to identify the enhancers and modulation of expression of the target genes. The Multi Assay Experiment (MAE) object was created with the methylation and expression data and separated into TP and NT samples. The distal enhancer probes were determined at a distance of 2 kilobases (kb) away from the Transcription Start Site (TSS) in the hypomethylated and hypermethylated (“hypo” and “hyper”) direction of TP. Distal probes, signifying regions enriched with enhancers, were identified by selecting 15,5378 probes based on the hg38 reference genome. An unsupervised method was applied to discern distal hypomethylated and hypermethylated probes. Methylation levels for each distal probe were systematically sorted across all samples in the TP and NT groups, with smoking status considered for each group. The methylation status of tumor samples was determined using stringent criteria (FDR < 0.01 and β-value > 0.3 or < -0.3). Inverse correlations between methylation levels of each probe and the corresponding gene expression were tested. The top 40% and bottom 40% of samples were classified as methylated and unmethylated groups based on methylation levels. Gene expression differences between these groups were analyzed using the Mann-Whitney U test to identify significant probe-gene pairs with negative correlations.

Further, the motif enrichment analysis of the distant enhancer probes was significantly differentially methylated and related to the target genes and was determined using the TF binding models from HOCOMOCO (V11)^17^. These models provided the input for Hypergeometric Optimization of Motif Enrichment (HOMER), an analytical tool that allowed the investigation of motif occurrences within a ± 250 bp range around each probe on HM450K and EPIC arrays illustrated with an Odd Ratio (OR) > 1 for each motif identified using a Fisher’s exact test and Benjamini-Hochberg correction^18,19^.

### Determining the overlapping DEGs

The overlapping DEGs were determined among the TCGA-LUAD, RNA-seq data of cigarette smoke-induced enhancers in A549 cells, and microarray data of the altered methylation of LUAD samples was determined using a Venn diagram (https://bioinformatics.psb.ugent.be/webtools/Venn/)^20^.

### Protein-Protein interaction network construction

The Search Tool for the Retrieval of Interacting Genes (STRING) was used to assess the interactions between DEGs with medium confidence at 5% significance. Cytoscape (V3.9.1) was used to visualize the network, and the cytoHubba plugin was used to identify the top 10 hub genes based on the Maximum Centrality Clique (MCC) algorithm^21,22^.

### Gene ontology enrichment analysis

The ShinyGO V0.77 (http://bioinformatics.sdstate.edu/go/) was used to perform the Gene Ontology (GO) enrichment analysis for the hub genes, identifying GO terms associated with biological processes, molecular functions, and cellular components, as well as groups of genes linked to high-level GO terms. The analysis was restricted to *Homo sapiens* and evaluated at a 5% FDR^23^.

### Survival analysis and expression patterns of hub genes

The Kaplan-Meier (KM) plot was used to evaluate the expression levels of hub genes related to overall survival. The tool generated KM survival plots that show the number of LUAD patients at risk based on the expression cohorts of genes over 200 months^24^.

### Protein expression levels of the hub genes in LUAD

To validate the protein expression levels of the hub genes in LUAD and normal LUAD adjacent tissues, immunohistochemistry (IHC) data were retrieved from the Human Protein Atlas (HPA, http://www.proteinatlas.org). The HPA was a resource for accessing IHC results pertaining to various proteins derived from proteomic analyses on cancer and normal tissues^25^.

## Results

### Differentially methylated sites

**Fig. 1:**
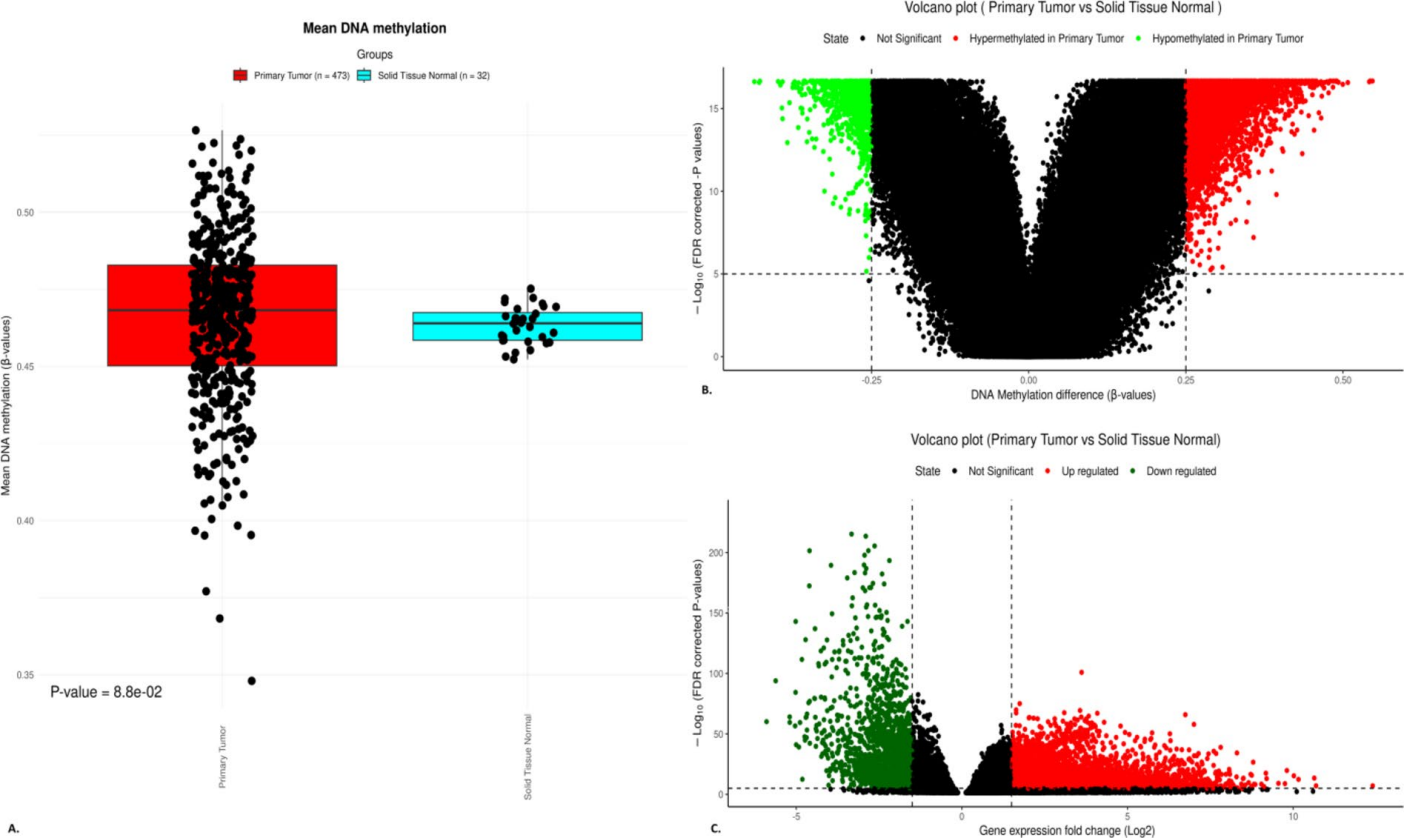
(**A**) The boxplot illustrates each sample’s mean DNA beta-methylation values. (**B**) The volcano plot illustrates the differentially methylated CpG sites. (**C**) The volcano plot illustrating the DEGs determined from the Transcriptome profile of the TCGA-LUAD data.

The mean of beta-methylation values of each sample in the TP and NT groups were determined using the box plot **(**Fig. 1A**)**. A genome-wide assessment revealed no statistically significant difference in the beta-methylation values among the groups (*p-*value 8.8 × 10^− 02^). This suggests that the methylation changes at specific regulatory regions are likely to play a critical role in modulating gene expression and driving tumorigenesis. Therefore, further analysis was conducted to assess the differential methylation at the CpG sites and their associations with target gene expression. The Wilcoxon test uncovered the differentially methylated CpG sites, comparing the two groups. The cut-off for the beta-methylation value was set as 0.25 at 5% FDR stringency. The analysis revealed 3,923 hypermethylated and 1,030 hypomethylated sites **(**Fig. 1B**)**. The cg08566455 probe was most hypermethylated with a mean difference of beta-methylation of 0.5469, and cg11201447 was the most hypomethylated site with a mean difference of the beta-methylation value of -0.4365.

### Differential gene expression

The adjacent genes to the CpG sites that exhibited differential methylation were identified, and their transcriptome profiles were analyzed. Genes that showed differential expression with the influence of altered DNA methylation were noted. The criteria for identifying DEGs were log2FC > 1 for upregulated and log2FC < -1 for downregulated genes, with an FDR rate of < 0.05 significance. Volcano plots (Fig. 1C) were generated to visualize the differential gene expression between the TP and NT groups using TCGA-LUAD transcriptome profiling data. The analysis revealed 1,901 DEGs with 1,095 upregulated and 806 downregulated genes.

### Enhancer linking by methylation/expression relationship

**Fig. 2:**
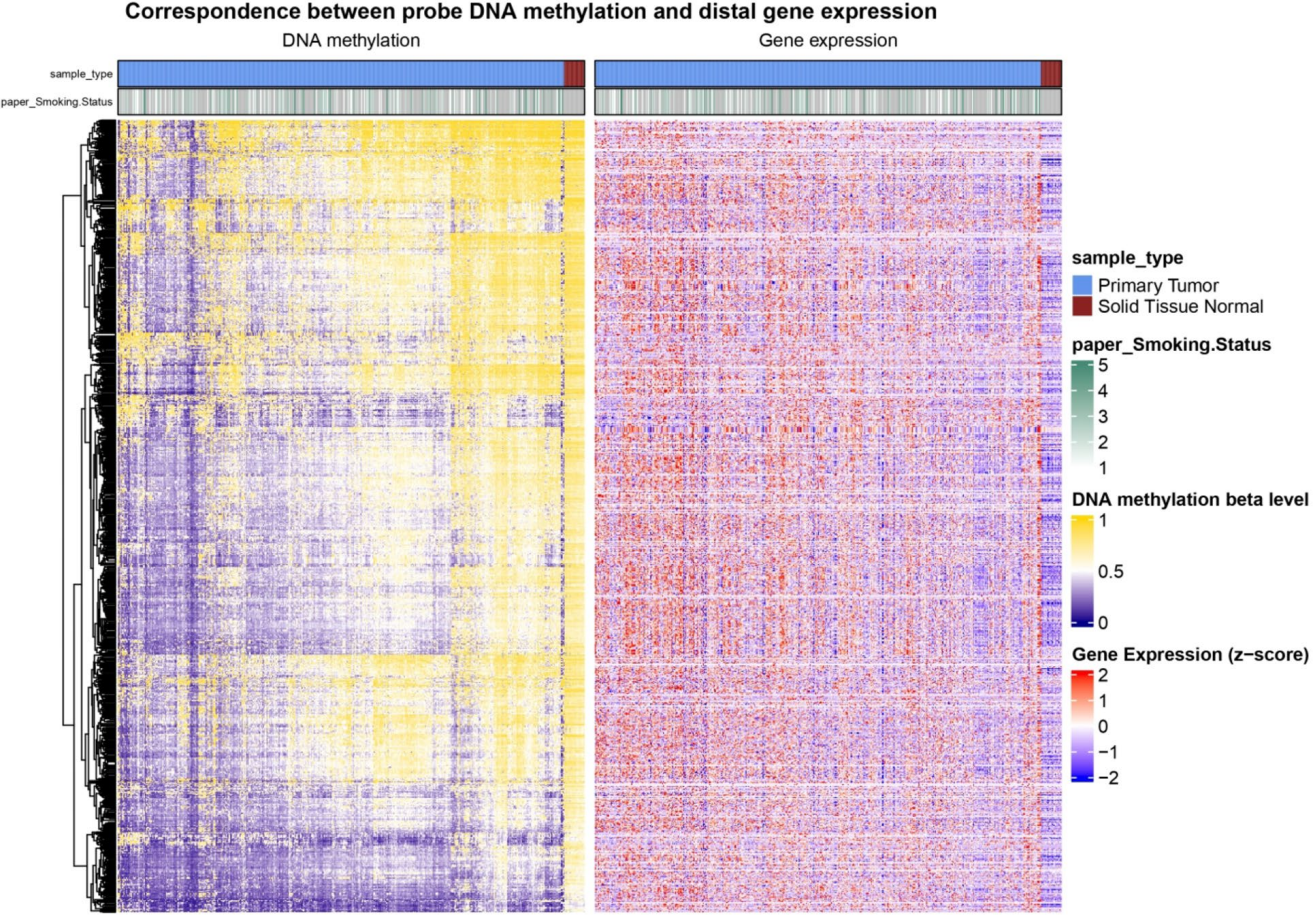
Heatmap illustrating the differential methylation of the enhancer probes comparing the TP and NT samples with their smoking status and the gene expression pattern of their target genes.

The ELMER analysis incorporated methylation and transcriptomic data from 473 primary tumor tissues and 32 normals within the TCGA-LUAD data. Subsequently, a heatmap illustrating the methylation and transcriptomic profiles was generated (Fig. 2). The Mann-Whitney U test was used to compare the gene expression levels between these groups, and 2,925 statistically significant probe-gene pairs exhibited negative correlations. From these probes, 250 bp base sequences upstream and downstream were extracted for motif mapping, resulting in the identification of 82 significantly enriched motifs from a total of 759 mapped motifs, exhibiting a minimum occurrence of 10 in the specified probe set and a lower threshold of 95% confidence interval for OR of 1.1 or higher. Among the distal probes corresponding to these motifs, differential classifications based on methylation levels further revealed significant relationships between methylation and TF activity, highlighting 1,638 TFs with differential expression between methylated and unmethylated groups, which aligned with the enriched motifs identified in the analysis.

### Common DEGs and network analysis

**Fig. 3:**
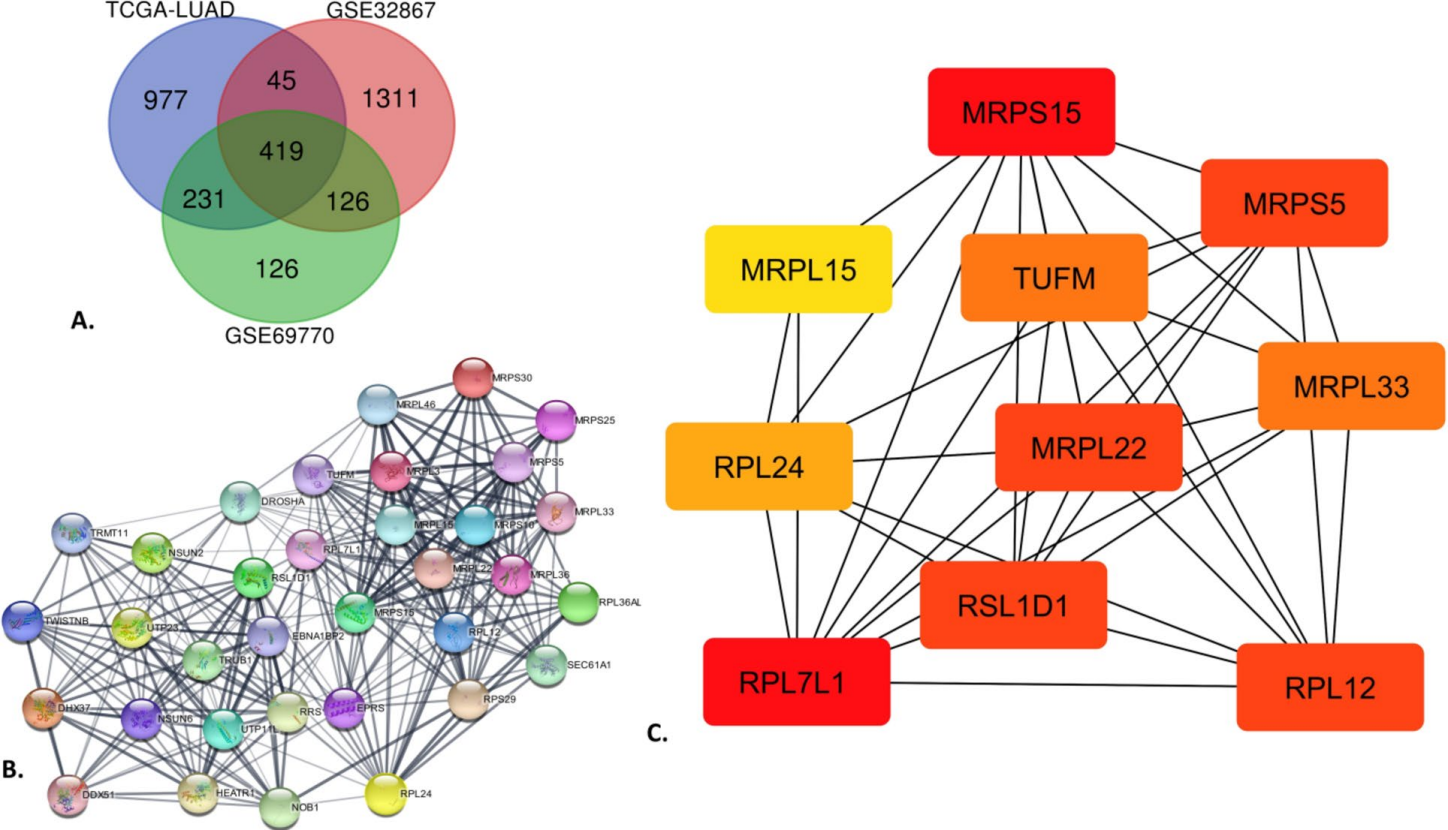
(**A**) Venn diagram of the compared DE protein-coding genes, excluding the TFs from the DEGs list from the three datasets, revealing 419 genes were differentially expressed, (**B**) Network topology analysis uncovered a top-scored cluster using MCODE and (**C**) The top 10 hub genes were identified using the MCC algorithm and ranked based on the node scores.

The expression of the identified target genes of the differentially methylated enhancer probes was validated by comparing them to the other datasets. The DEGs were identified based on the enhancer activation induced by cigarette smoke and an epigenome-wide smoke-induced DNA methylation profile of the LUAD cells (Supplementary Fig. S1). The total number of DEGs are listed in the Supplementary Table S1. The TFs from the list of DEGs were not included in determining overlap across the datasets and would develop a noise in the direct physical interactions analysis. The comparison was carried out using a Venn diagram. Figure 3A revealed that 419 protein-coding genes were differentially expressed in all three datasets. This piqued our interest in exploring these genes and determining their mechanisms and regulation in LUAD progression.

### Network analysis and enriched motifs of the hub genes

A static network-based technique was employed to study the physical and functional interactions between the DEGs (Supplementary Fig. S2). A notable cluster within the network was characterized by strongly connected portions, including significant genes. These genes were discovered to be important in the regulation of biological processes. The 419 DEGs were used to explore their functional relationships in “Homo sapiens” using a PPI network. The network comprised 419 nodes and 1,383 edges with a clustering coefficient of 0.266. The most densely interconnected region of the network was recognized as a significant cluster with the highest score of 16.667, comprising 34 nodes and 275 edges. The MCC algorithm uncovered the top 10 hub genes and ranked them based on node scores (Fig. 3B & C.). The differentially methylated sites, methylation status, and expression of the target hub genes are listed in Table 1.

**Fig. 4:**
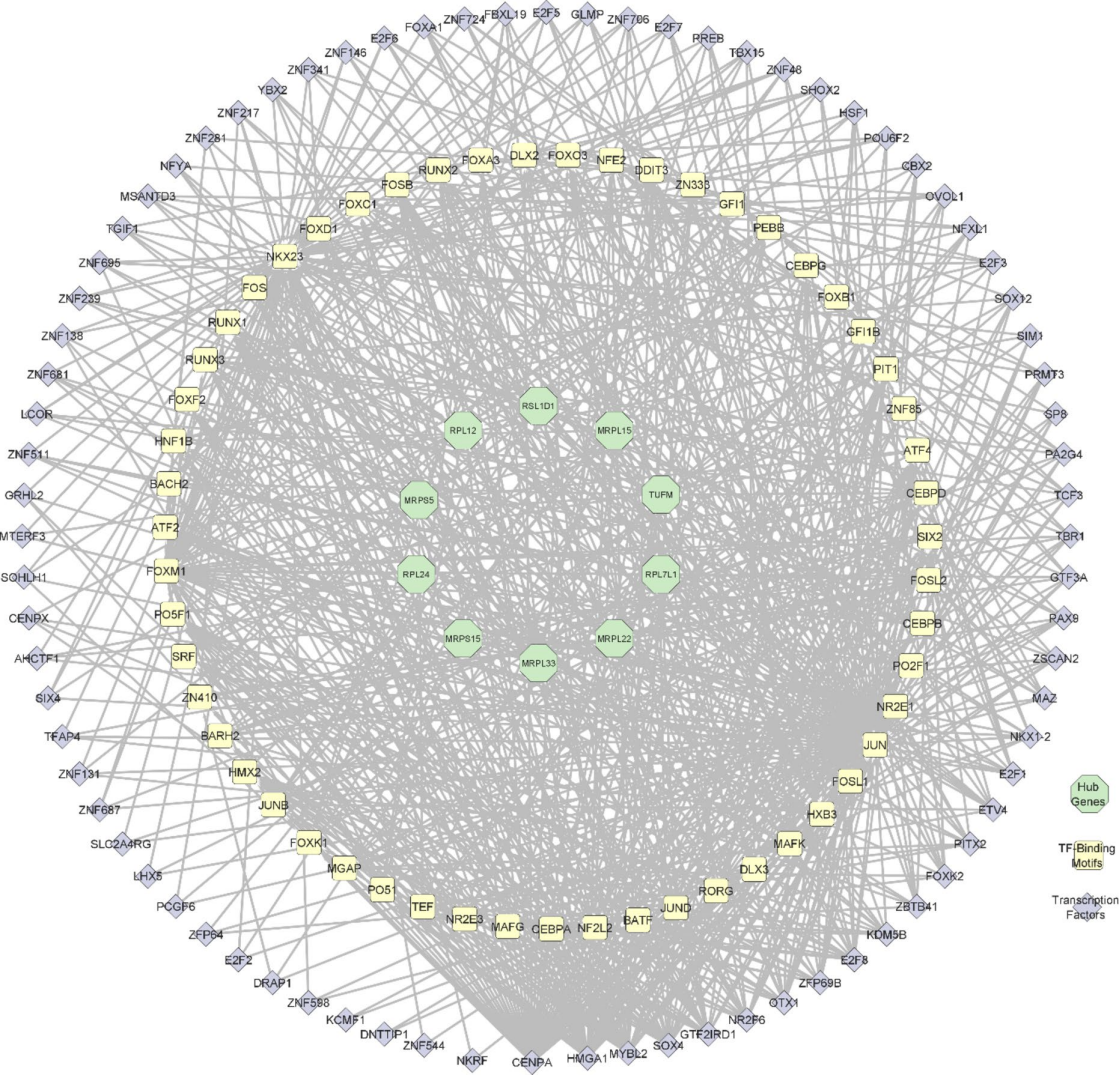
Enriched motifs with OR > 1.1 modulating the expression of hub genes identified using ELMER analysis.

The ELMER analysis revealed 82 motifs enriched to distal enhancer probes regulating the target genes in LUAD (Supplementary Material 2). The identified motifs regulating the hub genes in the progression of LUAD were determined, illustrated in Fig. 4. *JUN*, *NKX23*, *FOSB*, *RUNX3*, and FOSL1 were the top 5 enriched TF binding motifs to the hub genes and are associated with the dysregulation of the hub genes at the differentially methylated CpG sites (Table 2).

**Table 1:** The methylation status of the CpG sites, the corresponding target hub gene, and their expression profiles.

Hub genes	Differentially methylated CpG sites	Methylation status	Gene expression
*MRPS15*	cg16699316, cg19816734, and cg23814129	Hypomethylation	Upregulated
*MRPS5*	cg25820224, cg22592943, cg21665164, cg19280133, cg16176188, cg14086659, cg09496791, and cg01150454	Hypomethylation	Upregulated
*MRPL33*	cg27447296, cg21083314, cg18791923, cg18340214, cg18007524, cg14686798, cg12337011, cg06392451, and cg01055543	Hypomethylation	Upregulated
*RPL12*	cg10027509, cg10049880, cg13676383, cg13736810, cg13969579, and cg13981001	Hypermethylation	Upregulated
*RPL7L1*	cg00893987, cg03159127, cg09725975, cg16908031, cg19123639, and cg22909962	Hypermethylation	Upregulated
*RPL24*	cg25629615, cg19113686, cg16922785, cg16139664, cg12102538, cg11364645, cg10898607, and cg09243147	Hypermethylation	Upregulated
*MRPL15*	cg27026926, cg24665761, cg19897251, cg16889368, cg16129797, cg12096656, and cg10389722	Hypomethylation	Upregulated
*TUFM*	cg22973038, cg26925343, cg08335583, cg22973012, cg08230469, cg05970846, cg05666372, cg04459753, and cg04455064	Hypomethylation	Upregulated
*MRPL22*	cg05252892, cg07190450, cg13024389, cg15534020, cg17462962, cg20492933, cg22077101, and cg27295185	Hypomethylation	Upregulated
*RSL1D1*	cg02740093, cg08883955, cg16517021, cg20075675, cg22605342, and cg26999933	Hypomethylation	Upregulated

**Table 2:** Top 5 enriched TF binding motifs based on the degree in the TF-Motif-Hub gene interaction network.

Gene	Motif	Motif ID	Odd Ratio	*p*-Value	FDR
***JUN***		JUN_HUMAN.H11MO.0.A	3.34	9.06E-79	6.98E-76
***NKX23***		NKX23_HUMAN.H11MO.0.D	1.55	4.38E-06	2.56E-05
***FOSB***		FOSB_HUMAN.H11MO.0.A	3.18	2.14E-71	4.12E-69
***RUNX3***		RUNX3_HUMAN.H11MO.0.A	1.73	1.91E-14	2.59E-13
***FOSL1***		FOSL1_HUMAN.H11MO.0.A	3.19	1.79E-73	4.59E-71

### GO and functional enrichment

**Fig. 5:**
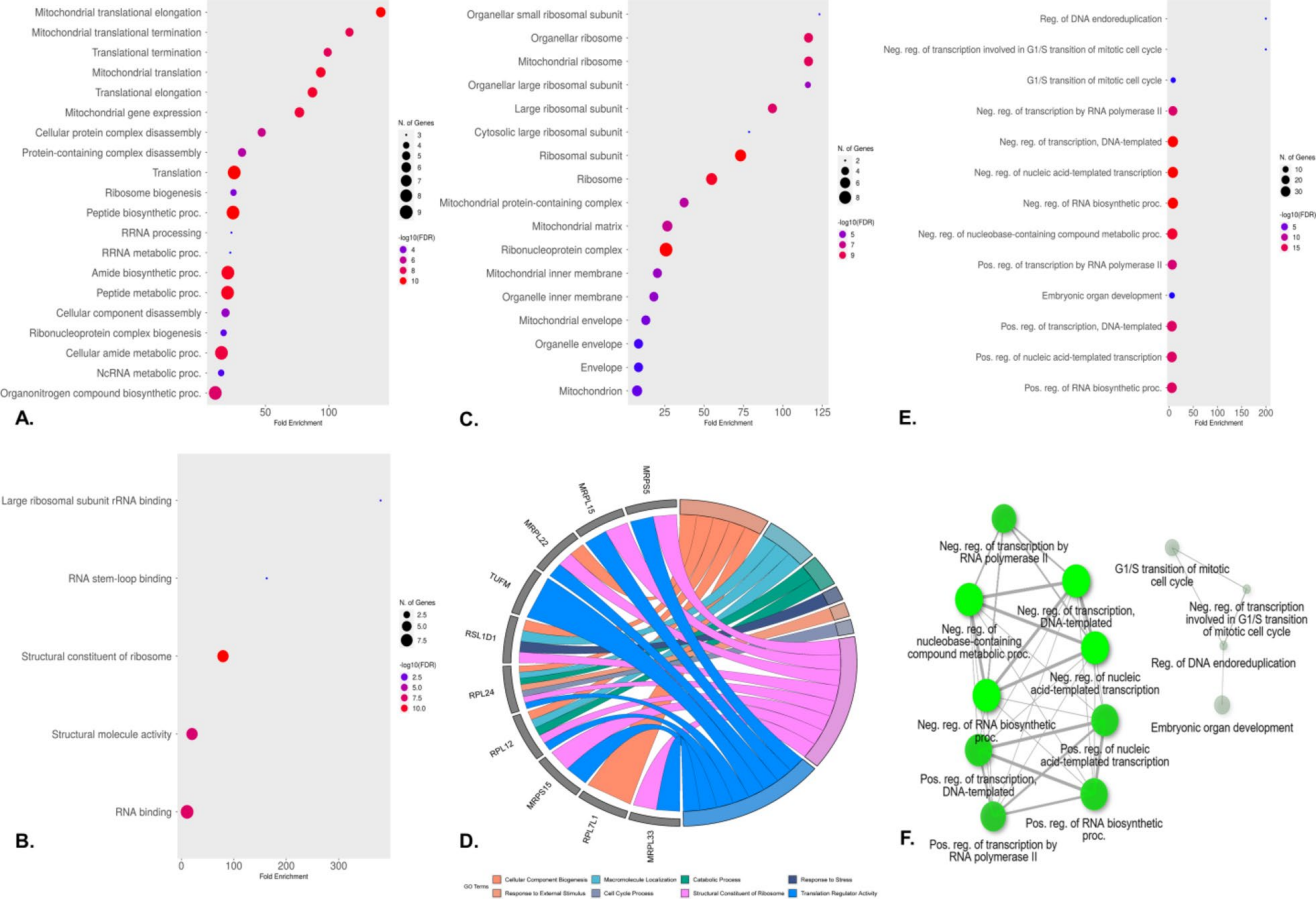
Gene functional enrichment analysis. (**A**) GO Biological Processes of hub genes, (**B**) GO Molecular Function of hub genes, (**C**) GO Cellular Components of hub genes, (**D**) The group of hub genes enriched to the high-level GO terms, (**E** &** F**). GO Biological processes of enriched TFs and the nodes representing enriched GO terms, with darker shades indicating higher enrichment significance and edges reflecting their functional relationships.

The biological processes associated with hub genes were investigated with a 5% FDR threshold. Hub genes exhibiting enrichment in GO terms were assessed based on total fold enrichment. These hub genes were enriched in biological processes and molecular functions, specifically involving translation elongation factors, impaired rRNA processing, and ribosome biogenesis, with mitochondria identified as the cellular component (Fig. 5A, B, **and C**). The analysis further revealed the top-ranked and predominantly enriched high-level GO terms through a chord plot to capture overarching functional categories and to provide a comprehensive understanding of the broader biological roles and processes influenced by the hub genes, encompassing cellular component biogenesis, response to stress, translation regulation activity, cell cycle processes, and the structural constituent of ribosomes (Fig. 5D). Additionally, the GO biological processes associated with TFs were delineated, highlighting enrichment in impaired regulation of transcription by polymerase II, other transcriptional regulations, and the G1/S mitotic cell cycle process (Fig. 5E, F).

### Clinical significance and validation

**Fig. 6:**
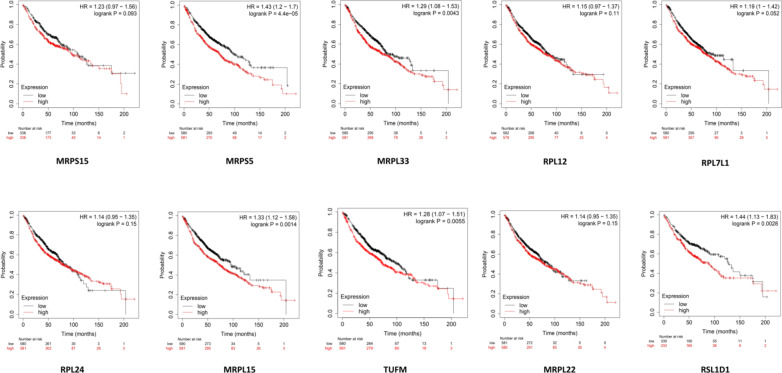
The clinical relevance of the hub genes is illustrated through KM plots of LUAD patients for 200 months.

**Fig. 7:**
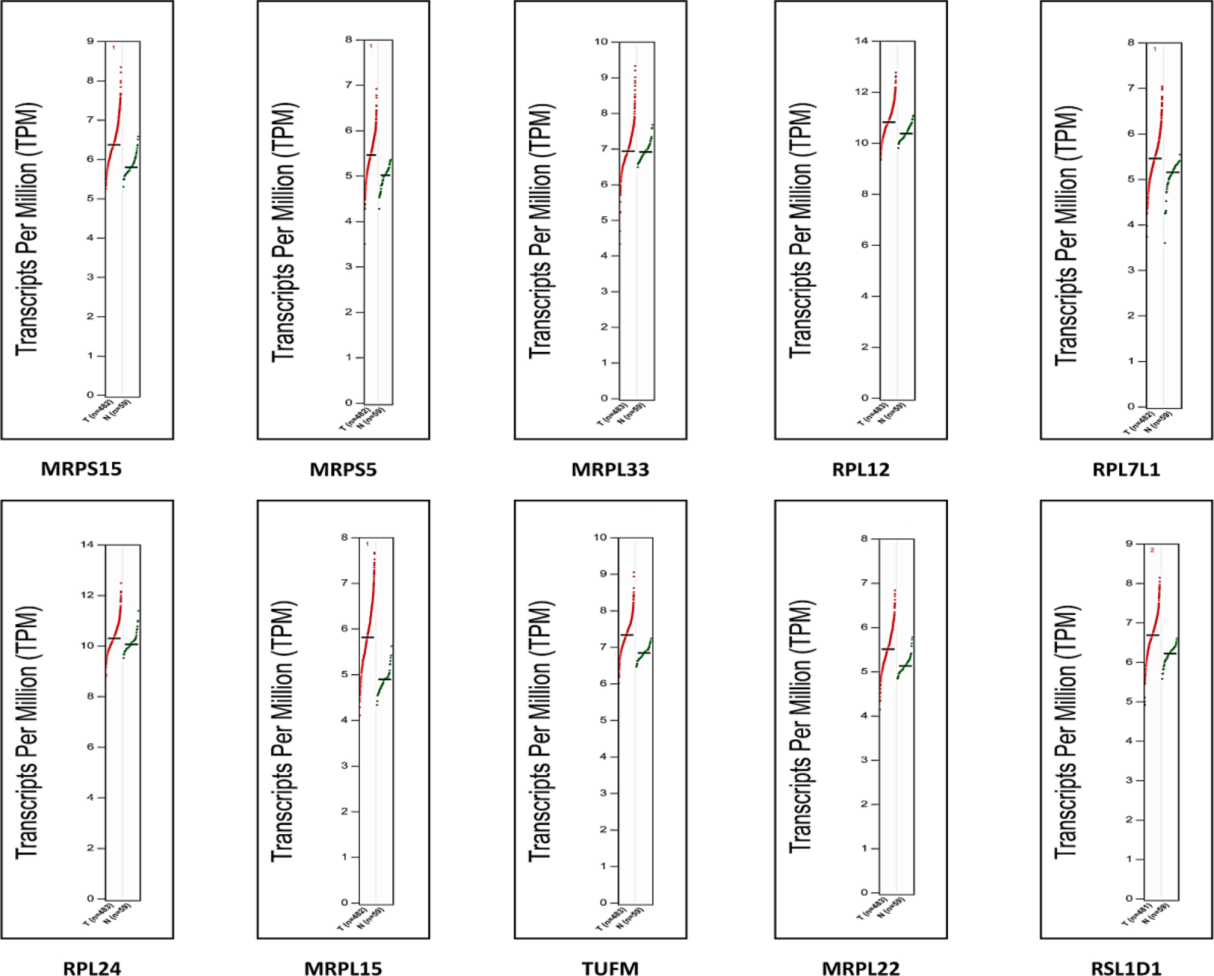
Dot plot illustrating the expression patterns of hub genes across LUAD tumors and their adjacent normal tissue samples. Each data point within the plot represents the gene expression in a specific sample, with red signifying tumor samples and green representing normal tissue samples.

The overall survival of expression cohorts in LUAD patients was analyzed for the hub genes over 200 months. The median survival between high and low-expression cohorts of specific hub genes in LUAD subgroups was assessed at a 95% confidence level using a log-rank *p*-value (Fig. 6). The KM plots demonstrated that expression cohorts of the genes were associated with LUAD pathogenesis, as indicated by a Hazard ratio greater than one. The survival analysis validated the log2FC expression patterns of the hub genes identified from transcriptome profiling. Ten hub genes that exhibited similar expression patterns in all three datasets and had validated expression cohorts in survival analysis were identified (Table 3). The expression patterns of the hub genes were also determined using the TCGA-LUAD RNA sequencing data converted to Transcripts per Million (TPM) at 95% significance. The TPM log2 (x + 1) elucidated the variance in the expression patterns between the LUAD patients and the adjacent tumor-normal samples (Fig. 7).

**Table 3:** The median expression duration, in terms of months, within the ordered cohorts of hub genes indicates OS in LUAD patients.

Genes	High expression	Low expression	Hazard ratio	log-rank *p*
*MRPS15*	96.2	108	1.23	0.093
*MRPS5*	71	99	1.43	4.4e-05
*MRPL33*	74	86	1.29	0.0043
*RPL12*	79.87	86	1.15	0.11
*RPL7L1*	95	80	1.19	0.052
*RPL24*	87.7	80	1.14	0.15
*MRPL15*	96	74	1.33	0.0014
*TUFM*	96.2	73	1.28	0.0055
*MRPL22*	86	79.87	1.14	0.15
*RSL1D1*	127	88.7	1.44	0.0028

### Protein expression levels of the hub genes

**Fig. 8:**
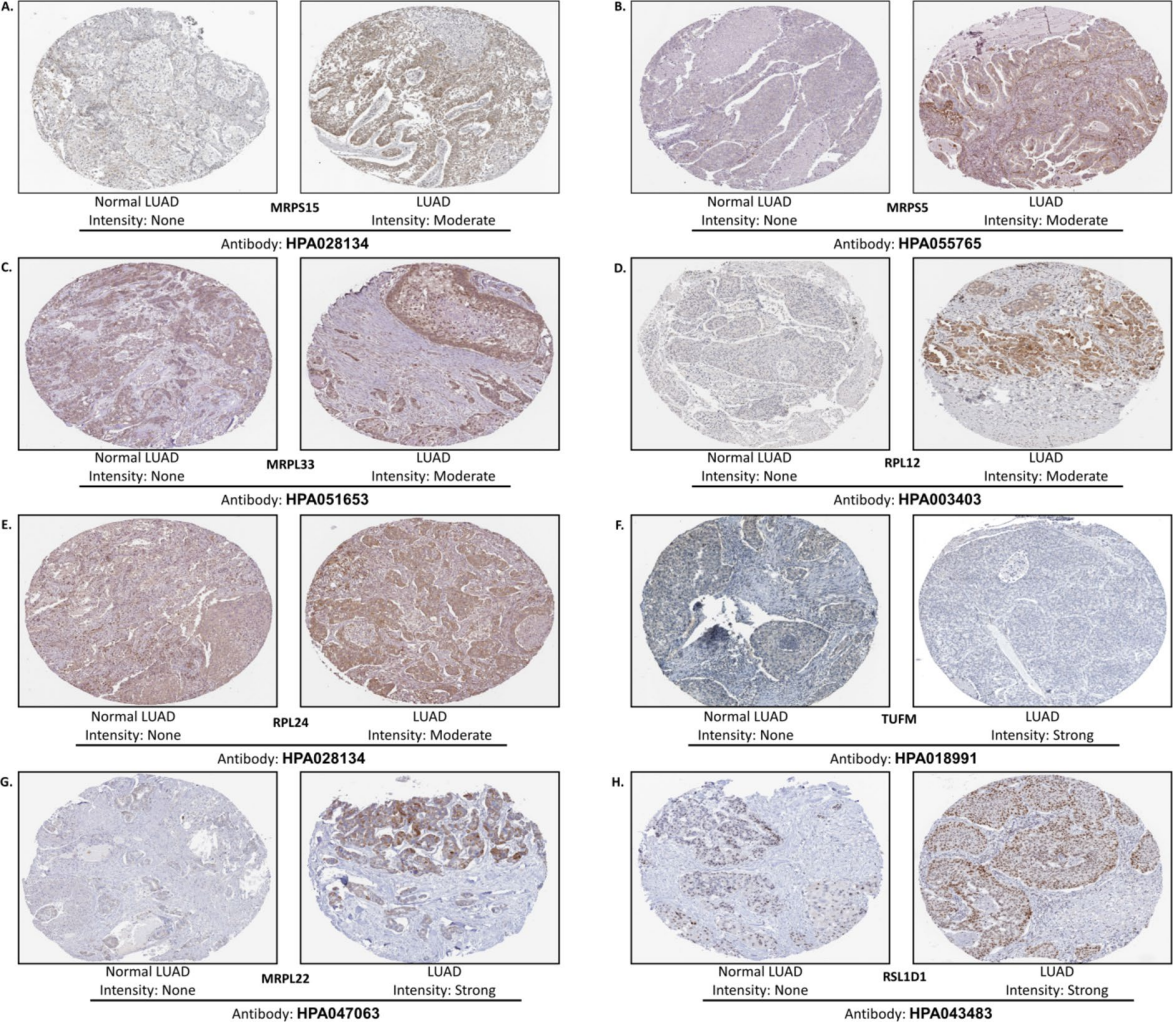
Protein expression profiles of the hub genes were determined using the HPA database.

The IHC outcomes pertaining to the protein expression of hub genes retrieved from the HPA database revealed distinctive patterns. Within the adjacent normal LUAD tissue, the IHC staining intensity for hub genes was consistently categorized as “None,” as depicted in Fig. 8. *MPRS15*, *MRPS5*, *MRPL33*, *RPL12*, and *RPL24* were moderately stained in LUAD tissue. Conversely, *TUFM*, *MRPL22*, and *RSL1D1* demonstrated a “strong” staining pattern within the LUAD tissue. Notably, information regarding the expression profiles of *RPL7L1* and *MRPL15* in LUAD tissue was unavailable in the HPA database. These findings contribute to a comprehensive understanding of hub genes’ differential protein expression profiles in the context of LUAD.

## Discussion

Lung adenocarcinoma represents one of the prevalent lung cancer and stands as a significant contributor to fatalities linked to cancer. The present comprehension of LUAD progression is not exhaustive due to its significant genetic diversity. Consensus in this field is still fragmented concerning the genetic changes responsible for the onset and progression of LUAD. The fusion of next-generation sequencing and sophisticated computational data analysis methods has transformed our comprehension of the genomic foundations of cancer mechanisms^26^. The synergy of epigenetic and transcriptomic data analysis is a potent tool for delving into the intricacies of cancer pathways. The absence of a significant global difference in beta methylation values between TP and NT samples highlights the complexity of LUAD epigenomics. It aligns with evidence that methylation alterations, particularly at distal regulatory regions, play a critical role in cancer pathogenesis^27^. Enhancers often exhibit region-specific methylation alterations that modulate transcriptional activity driving tumorigenesis^28^.

The TCGA-LUAD DNA methylation data analysis, stratified by smoking status and adjacent tissue normals, revealed distinct differential methylation patterns. These smoking-associated epigenetic alterations at specific CpG sites were associated with neighboring target genes, with differential gene expression, highlighting the critical role of DNA methylation in shaping transcriptional regulation and its contributions to LUAD pathogenesis.

According to recent studies, enhancers significantly impact the regulation of cell-specific attributes, which can result in alterations in disease-related gene expression patterns^29^. The ELMER analysis employed DNA methylation and gene expression data from the samples. The enhancers were identified by studying DNA methylation patterns and developed correlations between the status of enhancers and the expression levels of neighboring genes. The distal enhancer probes were identified, and probes lying beyond 2 kb regions around TSSs^18^. In addition, we identified potential target genes for differentially methylated distal probes using methylation and expression relationships. The correspondence between the methylated probes and the expression of the neighboring genes is illustrated in Fig. 2. The beta-methylation value 1 − 0 describes complete methylation and demethylation by comparing the TP and NT samples with their smoking status. Finally, 82 motifs were discovered and enriched to distal enhancer probes, which possessed significant differential DNA methylation regulating the target genes^30^. The intricate landscape of lung adenocarcinoma progression has been further illuminated by identifying the top 5 TF binding motifs through the degree of their interaction, each playing a distinct and pivotal role in the intricate regulatory dynamics that governed enhanced transcriptional activity.

Consequently, these motifs played a pivotal role in the modulation of target gene expression, fostering cell proliferation and survival through intricate signaling pathways in the tumor microenvironment^31–34^. Moreover, we assessed the expression profiles of each TF that were expected to bind to these motifs on enhancers. Our investigation revealed that the enriched TFs responsible for regulating enhancer-associated hub genes were predominantly associated with hypomethylated regions. The determined site-specific TF binding motifs exhibited enrichment within enhancers linked to potential target genes. Notably, these TFs were found to be engaged with enhancers combinatorially, collectively activating the expression of genes transcribed by RNA polymerase II^35^.

The PPI network assessed physical and functional relationships among DEGs^36^. Topology analysis identified significant, densely interconnected gene clusters, shedding light on crucial genes and their roles in regulating cancer processes induced by aberrant DNA methylation in LUAD patients^37^. The top 10 hub genes, crucial in cancer progression, were identified using the MCC centrality metric within the network’s largest cliques^37,38^. Gene ontology analysis identified GO terms describing their molecular functions, biological processes, and cellular components^39^. The GO terms associated with the hub genes were evaluated using a 5% FDR threshold. Total fold enrichment was calculated for the hub genes, which were determined using the topological analysis of the static PPI network model, demonstrating enrichment in GO terms. The analysis displayed paradoxically overexpressed RPs (*RPL24*, *RPL12*, and *RPL7L1*) and hypermethylated, enriched in processes related to impaired RNA processing and ribosome biogenesis^40–44^. An increasing number of comprehensive genomic investigations have found genetic abnormalities that lead to cancer by altering gene expression during both the transcriptional and post-transcriptional mechanisms, affecting numerous aspects of RNA processing^45^. Ribosomopathies are disorders linked with an increased risk of cancer caused by genetic abnormalities affecting ribosomal proteins, rRNA processing, and ribosome assembly. Ribosome biogenesis is a complicated process that unfolds in multiple stages from the nucleolus to the cytoplasm and is strictly controlled through multiple checkpoints and surveillance pathways. Overactive ribosome biogenesis can result from disruptions in these regulatory processes^46^. Ribosome biogenesis was active in a cell cycle-dependent manner, which was dysregulated by overexpressed *RSL1D1*, promoting cell proliferation^47,48^.

The mitochondrial protein translation, involving initiation, elongation, and termination, was driven by the overexpressed Mitochondrial Tu translation elongation factor (*TUFM*), which emerged as a prognostic marker, orchestrating with the MRPs, *MRPS15*, *MRPS5*, *MRPL15*, *MRPL33*, and *MRPL22*, for synthesizing essential mitochondrial proteins involved in OXPHOS complexes^49–51^. Hypomethylation disrupted the translation process, potentially affecting the assembly of OXPHOS complexes and, consequently, mitochondrial function. Mitochondrial dysfunction resulting from hypomethylation may lead to altered cellular metabolism, increased Reactive Oxygen Species (ROS) production, and a compromised electron transport chain, destabilizing the mitochondrial membrane potential (ΔΨm)^52,53^. Oxidative stress triggered by ROS exacerbated genomic instability and inhibited tumor suppressors^54,55^. The genomic instability, which may involve DNA damage responses, contributed to the dysregulation of the G2/M cell cycle checkpoint, facilitating the uncontrolled proliferation of cells^56^. These hub genes established a feedback loop that fueled the LUAD pathogenesis with metabolic reprogramming, cellular stress, and impaired cell cycle^57^.

**Fig. 9:**
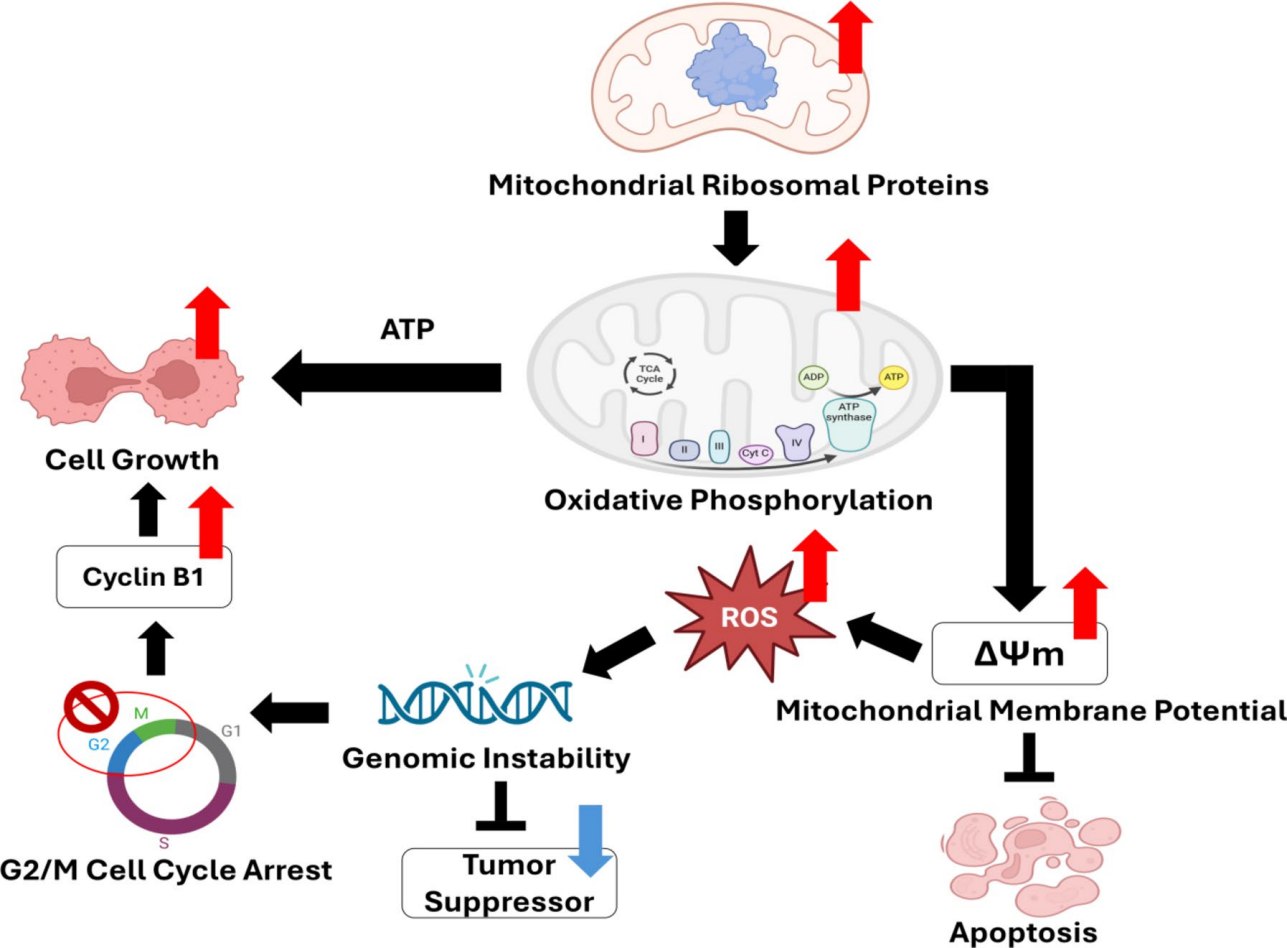
The working model illustrates the detailed sequence of events leading to LUAD progression, driven by the altered DNA methylation status of the enhancers regulating the expression of the target hub genes. Red arrows denote regions of elevated activity, while blue arrows highlight decreased activity.

Furthermore, the disruption of mitochondrial ribosomal proteins altered the balance between glycolysis and OXPHOS, favoring the Warburg effect. This metabolic shift might have provided the cancer cells support to the growth, as glycolysis generated energy rapidly and supported anabolic processes required for cell proliferation and metastasis^58^. The model (Fig. 9) depicts these comprehensive sequences of events within the intricate landscape contributing to LUAD progression.

It was crucial to identify biomarkers that can predict the clinical outcomes of the patients^59^. The OS of expression cohorts in LUAD patients was analyzed for the hub genes over 200 months^60^. The median survival between high and low-expression cohorts of specific hub genes in LUAD was assessed, demonstrating that the high-expression cohorts of the genes were associated with LUAD progression, as indicated by a Hazard ratio greater than one. The survival analysis validated the log2FC expression patterns of the hub genes identified from transcriptome profiling^61^. Ten hub genes that exhibited similar expression patterns in all three datasets and had validated expression cohorts in survival analysis were identified. The expression patterns of the hub genes were also determined using the TCGA-LUAD RNA sequencing data converted to TPM at 95% significance. The TPM log2 (x + 1) elucidated the variance in the expression patterns between the LUAD patients and the adjacent tumor-normal samples^62^. The mRNA expression levels of the central hub genes were assessed in LUAD tissues compared to normal tissues utilizing data from the HPA database. Most of the hub genes exhibited robust expression. A notable consistency in the expression patterns was observed at the mRNA and protein levels, underpinning the concordance between transcriptomic and proteomic data^63^.

This integrative approach allowed us to pinpoint critical regulatory occurrences, unveil promising therapeutic targets, and attain profound insights into the molecular foundations of LUAD. This holistic investigation paves the way for precision medicine strategies, enhancing the prospects of developing more potent treatments across diverse cancer subtypes. Moreover, additional experimental validations remain crucial to establish the significance of these findings.

## Conclusion

The study unveiled the complex molecular landscape of LUAD through an integrative epigenomic and transcriptomic analysis approach, shedding light on genetic diversity and providing a comprehensive understanding of tumor progression. Employing next-generation sequencing and advanced computational methods, we identified critical regulatory occurrences, RPs, and MRPs as potential therapeutic targets, paving the way for targeted therapeutics. DNA methylation and transcriptomic data analysis to enhancer dynamics and TF binding motifs were discerned using a robust ELMER technique. The hub genes derived using the static network model were associated with RNA processing, ribosome biogenesis, and impaired mitochondrial translation with differential methylation status, serving as potential biomarkers. Targeting these genes holds promise for novel therapeutic strategies and emphasizes the need for further experimental validation in advancing precision medicine for LUAD.

## Electronic supplementary material

Below is the link to the electronic supplementary material.

Supplementary Material 1

Supplementary Material 2

## Data Availability

The datasets used or analyzed during the current study are available from The Cancer Genome Atlas Database (TCGA-LUAD) and Gene Expression Omnibus (GEO) database (GSE32867 and GSE69770).

## References

[1] Siegel Mph, R. L. et al. Cancer Stat. 2023 CA Cancer *J. Clin.* **73**, 17–48 (2023).

[2] Thandra, K. C., Barsouk, A., Saginala, K., Aluru, J. S. & Barsouk, A. Epidemiology of lung cancer. *Contemp. Oncol.* **25**, 45 (2021). 10.5114/wo.2021.103829 PMC8063897 33911981

[3] Clark, S. B. & Alsubait, S. harvard. *StatPearls* (2020).

[4] Duruisseaux, M. & Esteller, M. Lung cancer epigenetics: from knowledge to applications. *Semin Cancer Biol.* **51**, 116–128 (2018). 28919484 10.1016/j.semcancer.2017.09.005

[5] Bakulski, K. M., Dou, J., Lin, N., London, S. J. & Colacino, J. A. DNA methylation signature of smoking in lung cancer is enriched for exposure signatures in newborn and adult blood. *Sci. Rep.* **9**, 1–13 (2019). 30872662 10.1038/s41598-019-40963-2 PMC6418160

[6] Petrovic, D. et al. Epigenetic mechanisms of lung carcinogenesis involve differentially methylated CpG sites beyond those associated with smoking. *Eur. J. Epidemiol.* **37**, 629–640 (2022). 35595947 10.1007/s10654-022-00877-2 PMC9288379

[7] Seiler, C. L. et al. Inhalation exposure to cigarette smoke and inflammatory agents induces epigenetic changes in the lung. *Sci. Rep.* **10**, 11290 (2020). 32647312 10.1038/s41598-020-67502-8 PMC7347915

[8] Zhang, Y. et al. Smoking-associated DNA methylation markers predict lung cancer incidence. *Clin. Epigenetics*. **8**, 1–12 (2016). 27924164 10.1186/s13148-016-0292-4 PMC5123284

[9] Fasanelli, F. et al. Hypomethylation of smoking-related genes is associated with future lung cancer in four prospective cohorts. *Nat. Commun.* **61** (6), 1–9 (2015). (2015). 10.1038/ncomms10192 PMC4682166 26667048

[10] Stueve, T. R. et al. Epigenome-wide analysis of DNA methylation in lung tissue shows concordance with blood studies and identifies tobacco smoke-inducible enhancers. *Hum. Mol. Genet.* **26**, 3014–3027 (2017). 28854564 10.1093/hmg/ddx188 PMC5886283

[11] Xiong, L. et al. Aberrant enhancer hypomethylation contributes to hepatic carcinogenesis through global transcriptional reprogramming. *Nat. Commun.* **10**, (2019). 10.1038/s41467-018-08245-z PMC6338783 30659195

[12] Colaprico, A. et al. TCGAbiolinks: an R/Bioconductor package for integrative analysis of TCGA data. *Nucleic Acids Res.* **44**, e71 (2016). 26704973 10.1093/nar/gkv1507 PMC4856967

[13] Selamat, S. A. et al. Genome-scale analysis of DNA methylation in lung adenocarcinoma and integration with mRNA expression. *Genome Res.* **22**, 1197 (2012). 22613842 10.1101/gr.132662.111 PMC3396362

[14] Ding, W., Kaur, D., Horvath, S. & Zhou, W. Comparative epigenome analysis using infinium DNA methylation beadchips. *Brief. Bioinform* **24**, (2023). 10.1093/bib/bbac617 PMC10147478 36617464

[15] Silva, T. C. et al. TCGA workflow: Analyze cancer genomics and epigenomics data using bioconductor packages. *F1000Research* 5, 1–55 (2016). 10.12688/f1000research.8923.2 PMC5302158 28232861

[16] Risso, D., Schwartz, K., Sherlock, G. & Dudoit, S. GC-Content normalization for RNA-Seq data. *BMC Bioinform.* **12**, 1–17 (2011). 10.1186/1471-2105-12-480 PMC3315510 22177264

[17] Kulakovskiy, I. V. et al. HOCOMOCO: expansion and enhancement of the collection of transcription factor binding sites models. *Nucleic Acids Res.* **44**, D116–D125 (2016). 26586801 10.1093/nar/gkv1249 PMC4702883

[18] Silva, T. C. et al. ELmer V.2: an R/bioconductor package to reconstruct gene regulatory networks from DNA methylation and transcriptome profiles. *Bioinformatics* **35**, 1974–1977 (2019). 30364927 10.1093/bioinformatics/bty902 PMC6546131

[19] Heinz, S. et al. Simple combinations of Lineage-Determining transcription factors prime cis-Regulatory elements required for macrophage and B cell identities. *Mol. Cell.* **38**, 576–589 (2010). 20513432 10.1016/j.molcel.2010.05.004 PMC2898526

[20] Zeng, Y. & Fan, R. Identification and verification of CCNB1 as a potential prognostic biomarker by comprehensive analysis. *Sci. Rep. 2022*. **121** (12), 1–12 (2022). 10.1038/s41598-022-20615-8 PMC9515086 36167975

[21] Cai, Y. et al. Identification of five hub genes as monitoring biomarkers for breast cancer metastasis in Silico. *Hereditas* **156**, 20 (2019). 31285741 10.1186/s41065-019-0096-6 PMC6588910

[22] Li, T., Gao, X., Han, L., Yu, J. & Li, H. Identification of hub genes with prognostic values in gastric cancer by bioinformatics analysis. *World J. Surg. Oncol.* **16**, 1–12 (2018). 29921304 10.1186/s12957-018-1409-3 PMC6009060

[23] Sekaran, T. S. G., Kedilaya, V. R., Kumari, S. N., Shetty, P. & Gollapalli, P. Exploring the differentially expressed genes in human lymphocytes upon response to ionizing radiation: a network biology approach. *Radiat. Oncol. J.* **39**, 48 (2021). 33794574 10.3857/roj.2021.00045 PMC8024183

[24] Liu, K., Kang, M., Li, J., Qin, W. & Wang, R. Prognostic value of the mRNA expression of members of the HSP90 family in non-small cell lung cancer. *Exp. Ther. Med.* **17**, 2657 (2019). 30930968 10.3892/etm.2019.7228 PMC6425268

[25] Zhao, P., Zhen, H., Zhao, H., Huang, Y. & Cao, B. Identification of hub genes and potential molecular mechanisms related to radiotherapy sensitivity in rectal cancer based on multiple datasets. *J. Transl Med.* **21**, 1–16 (2023). 36879254 10.1186/s12967-023-04029-2 PMC9987056

[26] Berger, M. F. & Mardis, E. R. The emerging clinical relevance of genomics in cancermedicine. *Nat. Rev. Clin. Oncol.* **15**, 353 (2018). 29599476 10.1038/s41571-018-0002-6 PMC6658089

[27] Aran, D., Sabato, S. & Hellman, A. DNA methylation of distal regulatory sites characterizes dysregulation of cancer genes. *Genome Biol.* **14**, 1–14 (2013). 10.1186/gb-2013-14-3-r21 PMC4053839 23497655

[28] Lemma, R. B. et al. Pioneer transcription factors are associated with the modulation of DNA methylation patterns across cancers. *Epigenetics Chromatin*. **2022 151 15**, 1–19 (2022). 10.1186/s13072-022-00444-9 PMC9016969 35440061

[29] Cho, J. W. et al. The importance of enhancer methylation for epigenetic regulation of tumorigenesis in squamous lung cancer. *Exp. Mol. Med.* 54, 12–22 (2022). (2021). 10.1038/s12276-021-00718-4 PMC8813945 34987166

[30] Wang, M. et al. Identification of DNA motifs that regulate DNA methylation. *Nucleic Acids Res.* **47**, 6753–6768 (2019). 31334813 10.1093/nar/gkz483 PMC6649826

[31] Chen, F., Liu, X., Bai, J., Pei, D. & Zheng, J. The emerging role of RUNX3 in cancer metastasis (Review). *Oncol. Rep.* **35**, 1227–1236 (2016). 26708741 10.3892/or.2015.4515

[32] Vallejo, A. et al. An integrative approach unveils FOSL1 as an oncogene vulnerability in KRAS-driven lung and pancreatic cancer. *Nat. Commun.* **2017 81 8**, 1–14 (2017). 10.1038/ncomms14294 PMC5321758 28220783

[33] Tang, C. et al. Abnormal expression of FOSB correlates with tumor progression and poor survival in patients with gastric cancer. *Int. J. Oncol.* **49**, 1489–1496 (2016). 27633497 10.3892/ijo.2016.3661

[34] Yu, W. et al. Genes regulated by Nkx2-3 in sporadic and inflammatory bowel disease-associated colorectal cancer cell lines. *Dig. Dis. Sci.* **55**, 3171–3180 (2010). 20165982 10.1007/s10620-010-1138-0

[35] Panigrahi, A. & O’Malley, B. W. Mechanisms of enhancer action: the known and the unknown. *Genome Biol.* 22, 1–30 (2021). (2021). 10.1186/s13059-021-02322-1 PMC8051032 33858480

[36] Suratanee, A. & Plaimas, K. Network-based association analysis to infer new disease-gene relationships using large-scale protein interactions. *PLoS One* **13**, (2018). 10.1371/journal.pone.0199435 PMC6021074 29949603

[37] Ashok, G., Miryala, S. K., Anbarasu, A. & Ramaiah, S. Integrated systems biology approach using gene network analysis to identify the important pathways and new potential drug targets for neuroblastoma. *Gene Rep.* **23**, 101101 (2021).

[38] Habib, I. et al. Differential gene expression and network analysis in head and neck squamous cell carcinoma. *Mol. Cell. Biochem.* **477**, 1361–1370 (2022). 35142951 10.1007/s11010-022-04379-3

[39] Thomas, P. D. The gene ontology and the meaning of biological function. *Methods Mol. Biol.* **1446**, 15 (2017). 27812932 10.1007/978-1-4939-3743-1_2 PMC6438694

[40] Kang, J. et al. Ribosomal proteins and human diseases: molecular mechanisms and targeted therapy. *Signal Transduct. Target. Ther.* 6, 1–22 (2021). (2021). 10.1038/s41392-021-00728-8 PMC8405630 34462428

[41] He, K. et al. Pan-cancer analysis of 60S ribosomal protein L7-Like 1 (RPL7L1) and validation in liver hepatocellular carcinoma. *Transl Oncol.* **40**, 101844 (2024). 38042135 10.1016/j.tranon.2023.101844 PMC10701367

[42] Islam, R. A. & Rallis, C. Ribosomal biogenesis and heterogeneity in development, disease, and aging. *Epigenomes 2023*. **7**, 17 (2023). 10.3390/epigenomes7030017 PMC10443367 37606454

[43] Bursać, S., Prodan, Y., Pullen, N., Bartek, J. & Volarević, S. Dysregulated ribosome biogenesis reveals therapeutic liabilities in cancer. *Trends Cancer*. **7**, 57–76 (2021). 32948502 10.1016/j.trecan.2020.08.003

[44] Popis, M. C., Blanco, S. & Frye, M. Posttranscriptional methylation of transfer and ribosomal RNA in stress response pathways, cell differentiation, and cancer. *Curr. Opin. Oncol.* **28**, 65–71 (2016). 26599292 10.1097/CCO.0000000000000252 PMC4805175

[45] Obeng, E. A. & Stewart, C. Abdel-Wahab, O. Altered RNA processing in cancer pathogenesis and therapy. *Cancer Discov*. **9**, 1493–1510 (2019). 31611195 10.1158/2159-8290.CD-19-0399 PMC6825565

[46] Elhamamsy, A. R., Metge, B. J., Alsheikh, H. A., Shevde, L. A. & Samant, R. S. Ribosome biogenesis: A central player in cancer metastasis and therapeutic resistance. *Cancer Res.* **82**, 2344–2353 (2022). 35303060 10.1158/0008-5472.CAN-21-4087 PMC9256764

[47] Prakash, V. et al. Ribosome biogenesis during cell cycle arrest fuels EMT in development and disease. *Nat. Commun.* 10, 1–16 (2019). (2019). 10.1038/s41467-019-10100-8 PMC6506521 31068593

[48] Ding, L. et al. Ribosomal L1 domain-containing protein 1 coordinates with HDM2 to negatively regulate p53 in human colorectal cancer cells. *J. Exp. Clin. Cancer Res.* **40**, 1–20 (2021). 34362424 10.1186/s13046-021-02057-8 PMC8344204

[49] Bao, S. et al. Potential of mitochondrial ribosomal genes as cancer biomarkers demonstrated by bioinformatics results. *Front. Oncol.* **12**, 835549 (2022). 35719986 10.3389/fonc.2022.835549 PMC9204274

[50] von Walden, F. et al. Reduced mitochondrial DNA and OXPHOS protein content in skeletal muscle of children with cerebral palsy. *Dev. Med. Child. Neurol.* **63**, 1204–1212 (2021). 34176131 10.1111/dmcn.14964

[51] Shi, H. et al. TUFM is a potential new prognostic indicator for colorectal carcinoma. *Pathology* **44**, 506–512 (2012). 22772342 10.1097/PAT.0b013e3283559cbe

[52] Lahtz, C. & Pfeifer, G. P. Epigenetic changes of DNA repair genes in cancer. *J. Mol. Cell. Biol.* **3**, 51 (2011). 21278452 10.1093/jmcb/mjq053 PMC3030973

[53] Raimondi, V., Ciccarese, F. & Ciminale, V. Oncogenic pathways and the electron transport chain: a dangeROS liaison. *Br. J. Cancer* 122, 168–181 (2019). (2019). 10.1038/s41416-019-0651-y PMC7052168 31819197

[54] Janic, A., Abad, E. & Amelio, I. Decoding p53 tumor suppression: a crosstalk between genomic stability and epigenetic control? *Cell. Death Differ. 2024*. **321 32**, 1–8 (2024). 10.1038/s41418-024-01259-9 PMC11742645 38379088

[55] Yu, W. et al. Reactive oxygen species bridge the gap between chronic inflammation and tumor development. *Oxid. Med. Cell. Longev.* 2606928 (2022). (2022). 10.1155/2022/2606928 PMC9256443 35799889

[56] Löbrich, M. & Jeggo, P. A. The impact of a negligent G2/M checkpoint on genomic instability and cancer induction. *Nat. Rev. Cancer*. **2007 711 7**, 861–869 (2007). 10.1038/nrc2248 17943134

[57] Benito-Lopez, J. J. et al. Partners in crime: the feedback loop between metabolic reprogramming and immune checkpoints in the tumor microenvironment. *Front. Oncol.* **12**, 1101503 (2023). 36713558 10.3389/fonc.2022.1101503 PMC9879362

[58] Burns, J. S. & Manda, G. Metabolic pathways of the Warburg effect in health and disease: perspectives of choice, chain or chance. *Int. J. Mol. Sci.* **18**, (2017). 10.3390/ijms18122755 PMC5751354 29257069

[59] Zhu, C., Menyhart, O., Gyorffy, B. & He, X. The prognostic association of SPAG5 gene expression in breast cancer patients with systematic therapy. *BMC Cancer*. **19**, 1–12 (2019). 31690268 10.1186/s12885-019-6260-6 PMC6833211

[60] Toledo, E., Epidemiological & Methods, N. *Prev. Cardiovasc. Dis. Through Mediterr. Diet.* 25–34 doi:10.1016/B978-0-12-811259-5.00002-0. (2018).

[61] Liu, C. et al. Survival-based bioinformatics analysis to identify hub genes and key pathways in non-small cell lung cancer. *Transl Cancer Res.* **8**, 1188 (2019). 35116861 10.21037/tcr.2019.06.35 PMC8797769

[62] Xie, Y. et al. Identification of hub genes of lung adenocarcinoma based on weighted gene Co-Expression network in Chinese population. *Pathol. Oncol. Res.* **28**, 1610455 (2022). 36032660 10.3389/pore.2022.1610455 PMC9399347

[63] Fagerberg, L. et al. Analysis of the human tissue-specific expression by genome-wide integration of transcriptomics and antibody-based proteomics. *Mol. Cell. Proteom.* **13**, 397 (2014). 10.1074/mcp.M113.035600 PMC3916642 24309898

