# Supplementary material for: Unraveling the intricate molecular landscape and potential biomarkers in lung adenocarcinoma through integrative epigenomic and transcriptomic profiling
Source: Sci Rep. 2025 Mar 17;15:9154. doi: 10.1038/s41598-025-93769-w (PMC11914463; doi:10.1038/s41598-025-93769-w)
Supplement: Supplementary file 1 — Supplementary Material 1 [file 41598_2025_93769_MOESM1_ESM.docx]

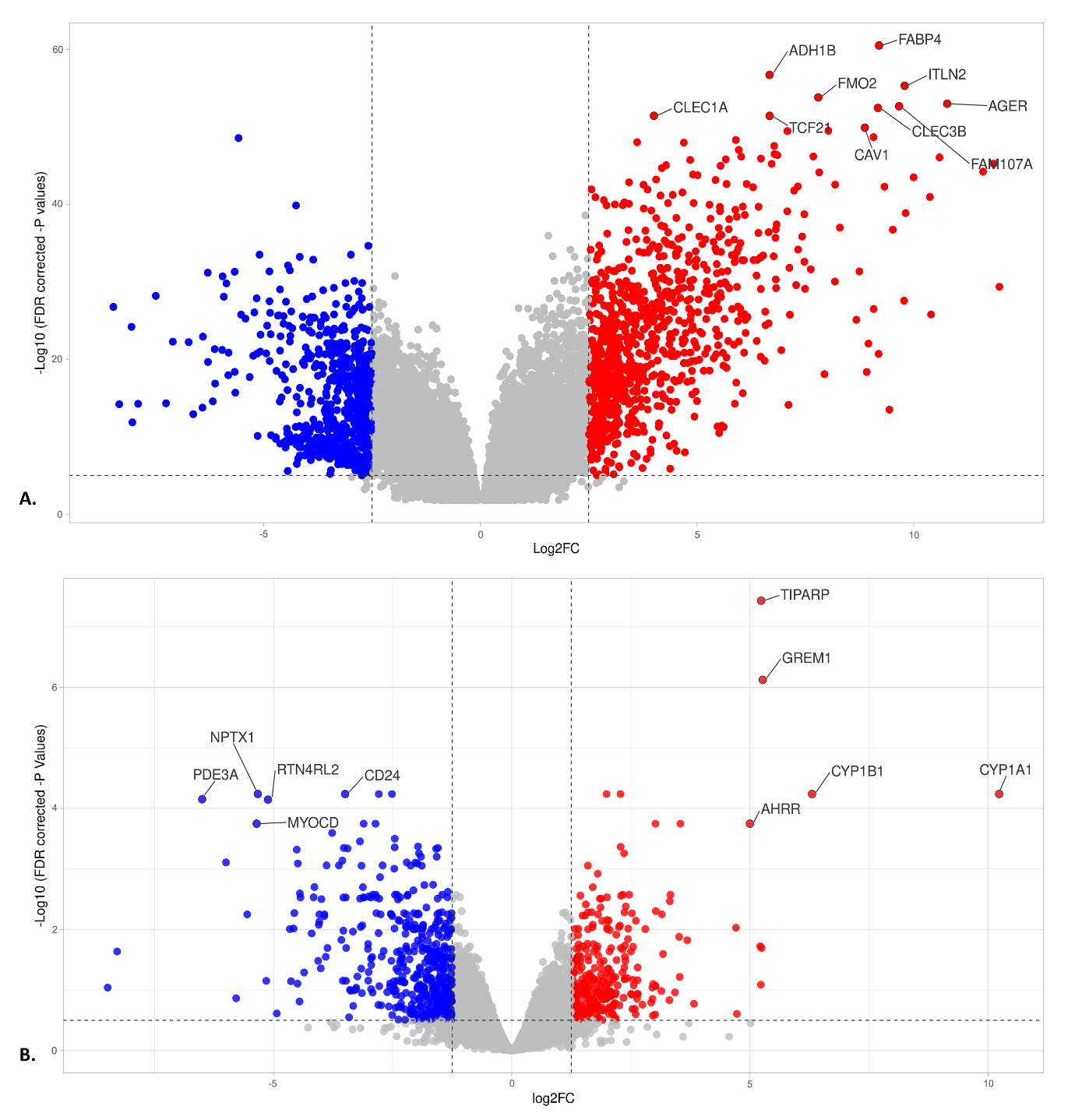


**Supplementary Figure S1.** Volcano plot illustrating the DEGs determined from the (**A.**) methylation profiling by array (GSE32867) and a (**B.**) RNA seq data of epigenome-wide profiling of DNA methylation in LUAD (GSE69770).

**Supplementary Table S1.** The total number of DEGs identified and their expression pattern in LUAD.

| **DATASET** | **TOTAL DEGs** | **UPREGULATED** | **DOWNREGULATED** |
| --- | --- | --- | --- |
| TCGA-LUAD | 1901 | 1095 | 806 |
| GSE69770 | 902 | 579 | 323 |
| GSE32867 | 1672 | 1037 | 635 |


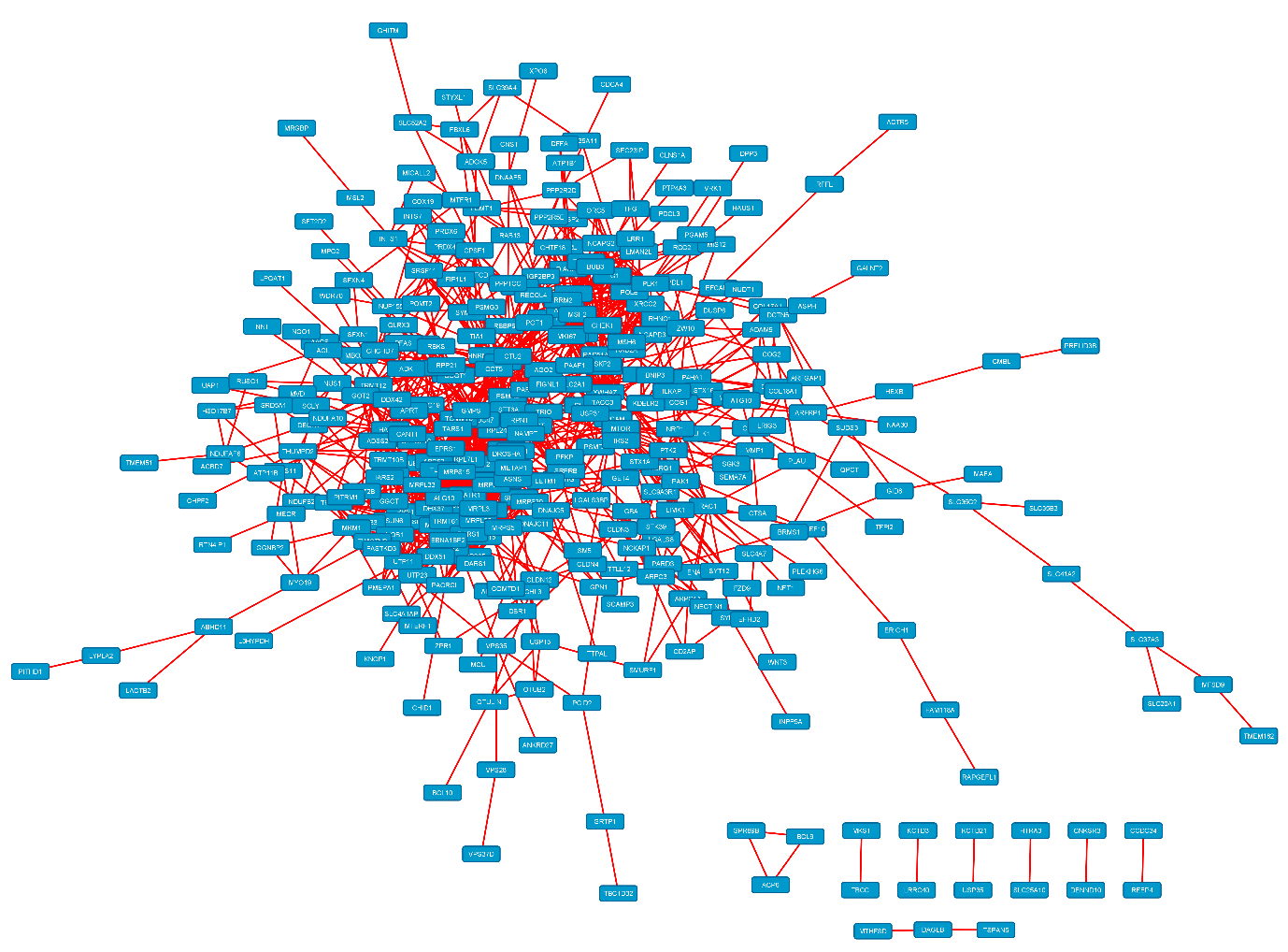


**Supplementary Figure S2.** A PPI network of 419 overlapping DEGs was generated using STRING.
