# Supplementary figures and images for: Unraveling the intricate molecular landscape and potential biomarkers in lung adenocarcinoma through integrative epigenomic and transcriptomic profiling
Source: Sci Rep. 2025 Mar 17;15:9154. doi: 10.1038/s41598-025-93769-w (PMC11914463; doi:10.1038/s41598-025-93769-w)

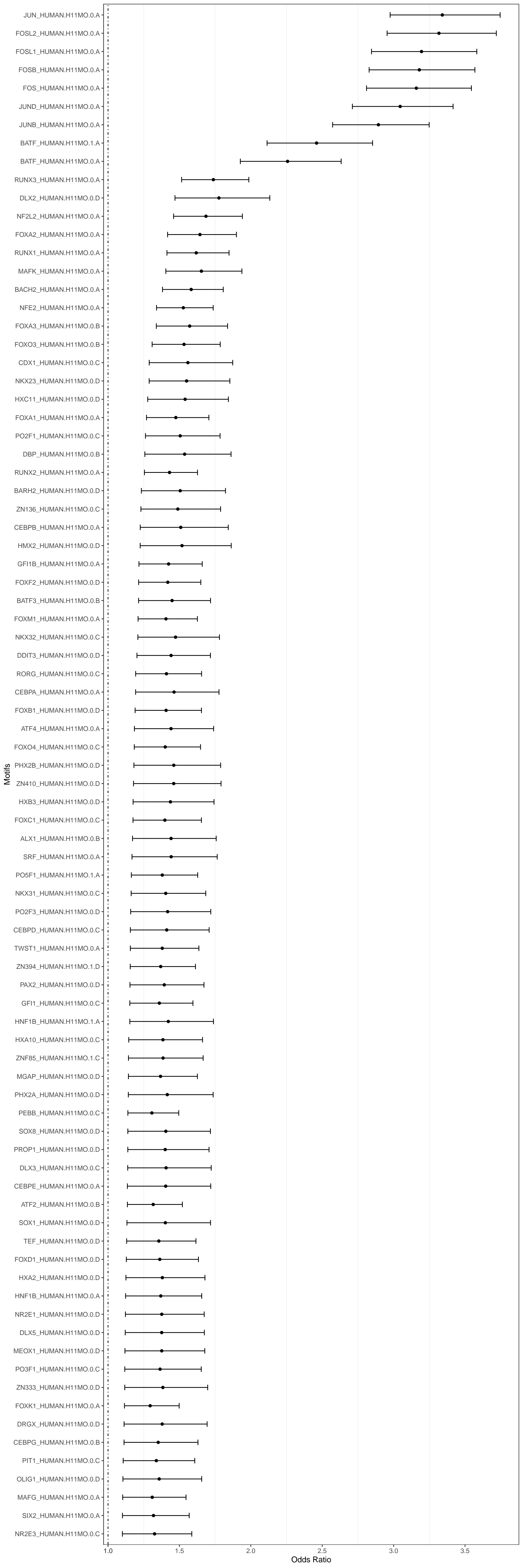

Supplement: Supplementary file 2 — Supplementary Material 2 [file 41598_2025_93769_MOESM2_ESM.pdf]
